# “I don’t know when he will be back”: life-changing events challenge the community ART Group model– a qualitative research study, Tete, Mozambique

**DOI:** 10.1186/s12889-021-12087-8

**Published:** 2021-11-04

**Authors:** Ivan Alejandro Pulido Tarquino, Emilie Venables, Rajá Reis Simone, Jose M. de Amaral Fidelis, Tom Decroo

**Affiliations:** 1 Médecins Sans Frontières, Maputo, Mozambique; 2 Southern Africa Medical Unit, Médecins Sans Frontières, Cape Town, South Africa; 3 Division of Social and Behavioural Sciences, School of Public Health and Family Medicine, University of Cape Town, Cape Town, South Africa; 4 Médecins sans Frontières, Tete, Mozambique; 5 OMES- PASSOS Project, Tete, Mozambique; 6 Health Provincial Directorate, Epidemiology Unit, Ministry of Health, Tete, Mozambique; 7 Institute of Tropical Medicine, Department of Clinical Sciences, Unit of HIV/AIDS & Infectious Diseases, Nationalestraat, 155 2000 Antwerp, Belgium; 8 Research Foundation Flanders, Brussels, Belgium

**Keywords:** HIV, Antiretroviral therapy, Community participation, Peer support

## Abstract

**Background:**

Since 2008 in Mozambique, patients stable on antiretroviral therapy (ART) can join Community ART Groups (CAG), peer groups in which members are involved in adherence support and community ART delivery. More than 10 years after the implementation of the first CAGs, we study how changes in circumstance and daily life events of CAG members have affected the CAG dynamic.

**Methods:**

A qualitative study using individual in-depth interviews (27) and focus group discussions (8) with CAG members and health care providers was carried out in Tete province, rural Mozambique. Purposive sampling was used to select participants. Data were transcribed and translated, and manual thematic analysis carried out to identify codes, which were then categorized in sub-themes and themes.

**Results:**

Data were collected from 61 CAG members and 18 health-care providers in 2017. The CAG dynamic was affected by life events and changing circumstances including a loss of geographical proximity or a change in social relationships. Family CAGs facilitated reporting and ART distribution, but conflict between CAG members meant some CAGs ceased to function. In some CAGs, the dynamic changed as pill counts were not carried out, members met less frequently or stopped meeting entirely. Some members did not collect ART at the facility when it was their turn, and others stopped taking ART completely. Health care providers were reported to push people living with HIV to join CAGs, instead of allowing voluntary participation. Some CAGs responded to adherence challenges by strengthening peer support through counselling and observed pill intake. Health-care providers agreed that strengthening CAG rules and membership criteria could help to overcome the identified problems.

**Conclusions:**

Changing life circumstances, changes in relationships and a lack of participation by CAG members altered the CAG dynamic, which sometimes affected adherence. Some CAGs responded to challenges by intensifying peer support, including to those diagnosed with virological failure. To ensure flexible implementation and modification of CAGs to the inevitable changes in life circumstances of its members, feedback mechanisms should be implemented between CAG members and the health-care providers.

**Supplementary Information:**

The online version contains supplementary material available at 10.1186/s12889-021-12087-8.

## Background

Striving to end AIDS by 2030, the World Health Organization (WHO) endorses the 90–90-90 UNAIDS targets: by 2020, 90% of People Living With HIV (PLWH) should know their HIV status. Of those, 90% should be retained on ART, and 90% of people on ART should have a suppressed viral load [1]. The vast majority of PLWH live in Africa. In 2016, Southern and Eastern Africa counted about 19.4 million PLWH, antiretroviral therapy (ART) coverage was around 60%, and about 50% of all PLWH were virally suppressed [2].

Various community-supported models showed high retention-in-care in different settings [3, 4]. In 2008 in Tete province in Mozambique, a community-based ART model was piloted [5]. Those stable on ART joined Community ART Groups (CAG), peer groups in which members take turns to pick up ART for their group at the health facility. Four-year retention in CAGs was 91.8% [6]. A key element of CAGs is that they are peer-led and based on social and/or geographical links between members [7].

Previous studies showed that life circumstances, such as social relationships, work and the distance between the patient’s home and the health facility affect long-term adherence [8, 9]. Moreover, peer support and higher levels of self-efficacy are recognised as enablers of sustained adherence to HIV care services [10–12]. However, to the best of our knowledge, no previous study has assessed how changes in life circumstances affect peer support in long-lasting peer groups.

More than 10 years after the implementation of the first CAGs in Tete, questions arise as to whether the peer-led CAG dynamic is sustainable in the long term. We were particularly interested to understand how changes in circumstance and daily life events of CAG members may affect the dynamic of this peer-led ART delivery model. We collected data from CAG members to show their experiences and perceptions, and from health care providers involved in CAG care, as they witnessed how the dynamic within different CAGs evolved over time.

## Methods

### Design

This qualitative research study explored adherence strategies amongst HIV patients on ART with virological failure in Changara-Marara district, Tete province. Findings with regards to perceptions and experiences of virological failure among CAG members and patients in regular individual care have been published elsewhere [13]. We developed topic guides, shown as supplementary file (supplementary file 1). For the present objectives we used data collected during individual in-depth interviews (IDIs) and Focus Group Discussions (FGDs) conducted with CAG focal points (informal group leaders), CAG members with a history of virological failure and health care providers in Changara-Marara district.

### Setting

In 2015 (a few years before data collection in 2017) a national prevalence survey was conducted. In Tete province, about 80% of all survey participants had ever been tested for HIV. The HIV prevalence among adults (15–49 years old) was 5.2% in Tete province, which was substantially lower than the national prevalence of 13.2% in Mozambique. In Tete province, among HIV positive survey participants, about 40% knew their serostatus, about one in three was on ART, and about one in five was virologically suppressed. The survey did not show separate data for the rural districts of Changara and Marara [14]. Changara counts 123,056 inhabitants, with 21 inhabitants per square kilometre. Changare has 11 health facilities, all with ART. Marara has a population of 73,044, with 27 inhabitants per square kilometre. Marara has 6 health facilities, of which 5 provide ART. More recently, Ministry of Health reports show that 79% of all estimated adult PLHIV were on ART in 2020. However, only 18% of PLHIV on ART had a viral load test. Of these, 64% were virologically suppressed.

Tete province is the heart of the country’s new coal mining industry, an industry that brought many job opportunities to people living in communities that previously depended on subsistence farming.

### ART delivery through CAGs

Patients in individual care come to the clinic monthly to collect their ART refills. Those stable on ART (> 15 years old, more than six months on ART and a CD4 > 350 cells/μl) are eligible to join a CAG [5]. Since viral load (VL) monitoring was introduced in 2013, [15] patients need to have a VL < 1000 cp/ml to join a CAG. Based on social and geographical affinity, those who are interested and eligible can join a group. CAGs are promoted at health facilities in waiting rooms, clinical consultations and counselling sessions.

CAG members take turns to collect ART for the rest of the group at the health facility on a monthly basis. Before going to the health facility a pill count is organised (this is considered as a measure of adherence, in which the remaining ARVs are counted before collecting refills). At the health facility, the group member representing the CAG has a clinical consultation, shares any important information about other members, and receives a treatment refill for all the members of his or her CAG. Upon returning to the community, they distribute the ART to the rest of the group. Each CAG chooses a focal point, a spokesperson responsible for the running of the group. Focal points and members are not remunerated [5]. Family CAGs were also formed to minimize the risk of conflict and facilitate ART distribution (Table 1).

**Table 1:** Types of Community ART Groups implemented in Changara and Marara districts during the study period, in 2017

CAG Type	Participant characteristics	Number of participants	Frequency of meetings	Place of CAG meeting
*Classic*	All participants are stable and active on ART	6	monthly	Community of origin
*Family*	All participants are stable and active on ART and are family members living in the same household	2–6	monthly	Household

*ART* Antiretroviral treatment, *CAG* community ART group

Patients with a detectable VL are also enrolled in enhanced adherence counselling (EAC). EAC uses a problem-focused counselling approach and consists of a minimum of two counselling sessions, organized at monthly intervals. EAC sessions aim to identify adherence barriers and ways to overcome them.

### Study participants and data collection

Study participants were purposively sampled from the following population groups: 1) health care providers, 2) CAG focal points and 3) and CAG members. Health care providers included clinicians, nurses and counsellors. Clinicians and nurses were part of the Ministry of Health (MOH) workforce. Counsellors were recruited and trained by a NGO (Médecins Sans Frontières; MSF), but were working in MOH-led health facilities and they were managed by both the counsellor supervisor (from the NGO) and the director of the health facility (from MOH).

For CAG members, purposive sampling was to select those who had experienced virological failure. CAG focal points were eligible if at least one member of their CAG had experienced virological failure. Eligibility was first assessed in the routinely updated HIV cohort database. A range of patients were selected from seven different communities within two different districts (Changara and Marara) to ensure that potentially diverse views, perspectives and experiences were included and presented. Trained HIV counsellors contacted CAG members and CAG focal points by phone or face-to-face. Health care providers were eligible if they were involved in HIV care provision.

After receiving an invitation to participate in the study from a counsellor, potential participants were informed about the aims of the study and what their participation would involve. Those who agreed to join the study were invited to sign an informed consent form. Taking into consideration privacy, data collection took place in venues in the community identified by the participants. FGD participants were offered refreshments and reimbursement for their transport costs if the venue was not within walking distance.

Twenty-seven IDIs and eight FGDs were conducted between August and September 2017, ten years after CAGs were implemented. The research team consisted of two non-Mozambican researchers, a Mozambican research assistant, a Mozambican translator who spoke Nyungwe, the local language and Portuguese and a notetaker. Topic guides with open-ended questions and probes were used during IDIs and FGDs (supplementary file 1).

FGDs were held to explore themes common to different groups of participants. More sensitive or personal issues were explored in individual IDIs. In-depth interviews and FGDs were organised in a location chosen by the participant, taking into consideration privacy and ease of access. Individual in-depth interviews and FGDs with CAG members were conducted in churches, school classrooms, private homes and meeting halls. People with similar roles were grouped together in the FGDs where possible. Separate FGDs were subsequently organised for physicians, nurses and pharmacists; HIV counsellors and CAG focal points. FGDs with healthcare providers took place in the health facility in the afternoon, when all clinical activities had finished. Groups were not divided by gender as this was not deemed to be necessary in this context. Participants were given refreshments and their transport costs were reimbursed.

Interviews were audio-recoded, transcribed verbatim and then translated in Portuguese (if the IDI or FGD was conducted in Nyungwe) by the person who had moderated the interview. The transcripts were verified by the other members of the research team. After each interview or FGD the research team discussed the collected data and if saturation had been reached. This was based on whether or not any new themes were emerging during data collection and if any further probes needed to be used.

### Data analysis

Thematic analysis was carried out independently by two Portuguese speaking co-investigators who discussed any discrepancies and differences in approach throughout the interim and final analysis. Coding and analysis were carried out manually. The transcripts were coded inductively, “ground-up” from the data. Codes were categorized into themes and sub-themes and organised in a coding tree [16]. Data were triangulated between the different groups participating in the study. Emerging themes were explored further during subsequent IDIs and FGDs. The co-investigators were experienced in implementing and evaluating community participation for HIV care. After analysis, selected quotes were translated from Portuguese into English.

### Ethics approval

Ethics approval was granted by the MSF Ethics Review Board, Geneva, Switzerland and the *Comité Nacional de Bioética para a Saúde* in Mozambique. Written informed consent was obtained from all participants. Participants were addressed by a codename during the FGDs to prevent them from being identified by others in the group. In addition, no names were documented.

## Results

Of 63 invited CAG members, 61 participated. Two female CAG members invited for individual IDI (one from Marara and one from Changara) did not want to participate after the information sheet was read to them due to concerns over time. Among those invited to participate in the FGDs, none refused to participate. Data were collected from 61 CAG members and 18 health-care providers. Of the 61 CAG members who took part, 27 participated in an IDI and 34 in an FGD. Eighteen health-care providers participated in three FGDs. Characteristics of study participants are given in Table 2.

**Table 2:** Characteristics of study participants involved in CAGs, from Changara and Marara districts in 2017

	CAG members		Health care providers	
	*n* = 61		*n* = 18	
*Female, n (%)*	43	(70.5)	10	(44.4)
*Age in years, median (IQR)*	46	(12)	34	(12)
*Participated in an in-depth interview, n (%)*	27	(44.3)	0	(0.0)
*Participated in a focus group discussion, n*	34	(55.7)	18	(100.0)
*Community*^*a*^ *(district)*				
*Matambo (Marara)*	9	(14.8)	2	(11.1)
*Marara Centro (Marara)*	8	(13.1)	4	(22.2)
*Marara Cachembe (Marara)*	9	(14.8)	2	(11.1)
*Missawa (Changara)*	8	(13.1)	2	(11.1)
*Mazoe (Changara)*	8	(13.1)	2	(11.1)
*Changara (Changara)*	10	(16.4)	3	(16.7)
*Dzunga (Changara)*	9	(14.7)	3	(16.7)

*IQR* interquartile range, *CAG* community ART group, *n* number

^a^Community = agglomeration, can be a village or a small city

As shown below, the CAG dynamic was affected by life events and changing circumstances, such as a loss of geographical proximity to other members or the health facility, or change in social relationships. Family CAGs facilitated reporting and pill distribution, but the group could cease to function if there was conflict within a relationship. In some CAGs, the dynamic changed as pill counts were not carried out, members met less frequently or stopped meeting entirely before sending a representative to the clinic. Some members did not collect ART at the facility when it was their turn, and others stopped taking ART altogether. CAG members reported that health-care providers made membership of a CAG obligatory, instead of promoting voluntary participation. Some CAGs responded to adherence challenges by strengthening peer support through counselling and observed pill intake. Providers agreed that strengthening CAG rules and membership criteria could overcome these identified problems.

### Events or circumstances affecting CAG dynamics

All participants agreed that a well-functioning CAG with a strong bond between members is built on social and geographical proximity: *“each neighbourhood organizes its own groups, this is how we organize”* (IDI29, female CAG member).

#### Loss of geographical affinity

CAG functioning can become challenging if members migrate to larger, more urban areas such as the city of Tete: *“There, in our neighbourhood, some groups existed, but they dissolved ( …*) *There was an exodus of people, many went to the city.”* (IDI 8, male CAG member)*.* Working in the mines in particular was reported to interfere with being an active and engaged CAG member as this involved longer periods away from home.

Some CAG members travelled for employment, but were still classified as belonging to CAGs by health facility staff, as this health care provider explained:

“*The economic situation faced by the population makes things difficult. A CAG member wants to go to the mines, is there, but is [still] counted as being a CAG member ( …) sometimes he stays there for a long time without picking up his drugs ( …) I don’t know when he’ll be back. And when he’s there, he’s not taking his medication.*” (FGD 1, clinical officer).

On the other hand, CAG members assisted each other in collecting and distributing ART refills for short and unplanned trips, taking advantage of the flexibility that CAG membership allows: *“I can meet with my colleague [fellow CAG member] even on a Saturday.”* (IDI 16, female CAG member)*.*

CAG members also reported being able to borrow pills from each other if they had to travel unexpectedly, returning them to the member upon their return: *“If someone is preparing to travel before their next date to collect drugs, then he can ask another person for them ( …) this is amicable. Then, when he receives his [ART refill], he returns the medication.”* (FGD 6, CAG members)*.*

Some members decided to leave their CAG as their frequent travels did not allow them to fulfill responsibilities, such as representing the CAG at the health facility when it was their turn:

*“I have a date to pick up the drugs but I’m in the city ( …) I move around a lot, I’m a person who doesn’t stay in a fixed place ( …) they called me to say “we’re always the ones who pick up the drugs”. Leave me; I will stay by myself [leave the CAG].”* (IDI 39, female CAG member).

#### Loss of social affinity

Some members returned to individual clinic care because they had chosen to, or because it was suggested to them by the other members of their group. Participants reported how the composition of CAGs could change over time as members no longer got along with each other.

Sometimes even the CAG focal point was excluded from their group by the other members. *“We had a focal point, but he didn’t join our group anymore, he didn’t come, thus we put him aside ( …) the group functionned without him.”* (IDI 13, *female* CAG member).

Another cause of loss of social coherence within CAGs was alcohol use. Members known to consume large amounts of alcohol could be asked to leave a CAG, as reported by this interviewee, who believed that members who drank alcohol may then disclose the HIV status of others: “*When a person drinks, he doesn’t control what he says ( …) he starts saying things he should not mention, he starts talking about the group so we exclude people who drink.”* (IDI 29, female CAG member).

Alcohol use was also perceived as having serious consequences, with one person reportedly becoming ‘addicted when he was in the mines’ and subsequently dying.

#### Pregnancy

Pregnancy was reported as another life event causing female members to leave CAGs due to the need for increased medical follow-up, which in turn altered the group dynamic. Pregnant members returned to individual care because *“the [pregnant] person has to go monthly to the health facility, whereas in the CAG this is different, a person goes once, depending on the number of group members”* (IDI 33, female CAG member).

### Benefits and challenges with family CAGs

CAGs were also formed within families: “*we are in a group of four people; I am with my three sisters-in-law. We were five including my brother, but my brother just passed away, now we are four.”* (IDI 22, female CAG member), which was seen as beneficial because of the convenience of being in the same household. This was also supported by a health-care provider:

*“ … they live in the same backyard, so they sit there to check this matter, who took it [the pills], who did not take it”* (IDI 22) and *“you have direct control: you don’t have to do any effort to take the bottles and to call the people for a meeting”* (FGD 4, CAG focal points)*.*

However, other health-care providers suspected that adherence challenges were under-reported in family CAGs as members wanted to protect each other from criticism or judgement from a nurse.

*“A member came in the following month, and he collects pills for another [member], but he does not “tell the truth”. He does not say what is happening in the group, and the health care provider does not know whether or not he is not taking his treatment, or if he already left the CAG.”* (FGD 2, nurse)

Health care providers reported problems in a CAG after a couple joined a group and their relationship ended. *“One of the group members came to me with about four bottles [ART] of one the members of the group. (…) He said ‘well, I’m the husband of one the members of the group, we are divorced and unfortunately I haven’t been able to find her to give her the drugs for four months.’* (FGD 2, health care providers, nurse).

### Modifications to the CAG dynamic

Most CAG members who were interviewed reported that they met in the community, typically the day before the next scheduled ART refill date. Many members reported that they carried out a pill-count, shared experiences, counselled each other and reported problems to the nurses at the health facility: *“the day before picking up the refill, we meet to talk a little bit … we do the pill count ( …) we see who is taking it [ART] properly and who is not ( …) this helps us*.” (IDI 10, female CAG member).

#### Adaptations to CAG meetings and reporting

Many CAG members, however, reported adapting the typical CAG model and ‘rules’ to better fit with their circumstances. Some did not conduct a formal pill count:” *we don’t ask people to bring the bottles for verification [pill-count]: we just ask the person “how many pills are remaining?”* (IDI 13, female CAG member).

Other groups did not organise meetings for all CAG members together (a key part of creating a CAG), with the focal point making home visits to assess the adherence of members instead: *“We don’t come together. We are in a village, in a neighbourhood, and I, as focal point, go door-to-door. One day before the next refill, I take the CAG group card and have a look at the number of pills that are remaining.”* (FGD 4, CAG focal points)*.*

Sometimes members stopped participating in their CAG, and did not pick up their pills from the focal point in their community. Then the CAG representative can return to the health facility to return the pills to the nurse. Providers reported that those who represented the CAG at the monthly health-facility visit were not always well-informed about the whereabouts of the other members. According to providers, peer groups that were established many years ago had weakened and members rarely met in person to discuss treatment adherence. As the below examples show, health-care providers reported that some CAG members asked family members to distribute ART instead of meeting with other members themselves:

*“When that member stays there … he is making charcoal, or he is selling products along the street, then I didn’t manage [to meet them], then maybe I gave [the medication] to the son.”* (FGD 2, health care providers, clinical officer).

*“We meet in this season as there are no agricultural activities. But, in the season of agricultural activities each person wakes up to go to the field and returns tired so that’s why we don’t talk.”* (IDI 2, female CAG member).

Rotating representation of the CAG at the health facility was reported to be challenging by CAG members and health care providers, which also meant that there were some members who were not benefitting from viral load and CD4 monitoring. *“One or two go regularly to the health facility, and the other four, or three, five, might not come to the health facility. Their blood is not collected to do the CD4 analysis.”* (FGD 2, health care providers, nurse).

According to health-care providers, some members were still reported as being active in the group, even though they did not pick up their pills through the CAGs.

Some CAG members were reported as being ongoing CAG members, but did not take their pills. In one case, a CAG member died and medication was found in their home: “*She died and when we looked in her house we encountered these unopened bottles.*” (FGD 4, CAG focal points). Providers suspected that during pill counts such patients may “*take an old bottle and show it [the remaining number of pills], while the new bottle is kept aside”* (FGD 1, health care providers, nurses).

#### Pressure to join CAG

Some patients felt pressured to join a CAG by the nurse or by fellow patients, which resulted in CAGs lacking in a peer dynamic and bond: *“those from the clinic called me and told me that if did not accept [joining a CAG], that maybe they will not give me their medication”* (FGD 1, health care providers, nurse).

Sometimes patients in individual care are asked by the care providers to join a CAG without exploring the patient’s preferences: *“Haaaaa, it was them [care providers] who told me to join these people ( …) they did not explain the CAG. I believed that if they told me to join, I have to believe that the CAG is good for me.”* (IDI 9, male CAG member).

Moreover, some CAG members found it difficult to negotiate changes such as leaving one CAG to join another: “*Well, I cannot order the government and say: I don’t want this, I don’t want that ( …) I have no voice. But for me it would be good if I could change group.”* (IDI 5, female CAG member).

### Response to challenges

Some members identified non-adherence by observing the deterioration of a member’s health as ‘the first sign’: “*The first sign is a person with continuous health problems”* (IDI 16:128–129). On other occasions “*[lab] results arrive which show that a person has difficulties with taking [the medication]”* (IDI 16, female CAG member). CAG responses to identified non-adherence often included home visits to explore potential barriers. Some CAGs were able to adapt the social and peer dynamic to respond to such emerging needs:

*“When she was not adhering to her treatment [she said] ‘I don’t want this treatment anymore because I have many problems with my son, it’s better to stop taking medication so that I can die.’ But when I did the follow-up to improve adherence, she was able to say: “Yes, I’m adhering well with the treatment and with the pill count, also with the support of the group.”* (FGD 5, health care providers, counsellors:).

In cases where CAG members did not accept their HIV status, it became challenging to offer them peer support: “*as the person doesn’t feel sick, and sometimes thinks it [HIV] is an invention, it’s tough to accept, therefore it is difficult for a person who is ill to take medication as he feels healthy”* (FGD 3, CAG focal points).

Others wanted to be left alone, as reported by CAG focal points: *“This is my life, if I have to die, I will die alone, do not interfere.”* (FGD 3, CAG focal points).

Some CAGs such as the one cited here implemented direct observed therapy (DOT) to support each other: *“The group was always providing support, there is a neighbour who always takes the medication together, and now she’s fine.”* (FGD 5, health care providers, counsellors).

Such an intensified adherence support strategy can also involve all the members of the group as well as monitoring of the strategy: “In the group, we take turns *to support the person ( …*), *we stay there to observe [the pill intake], first goes this person, then goes the other ( …). After some time we go and have a look if the person returned to the normal routine, and then we leave the person taking the medication by himself.”* (IDI 16, female CAG member)*.*

Providers felt that they should apply more rigorously pre-established criteria when forming CAGs. Moreover, they proposed to review the composition of the CAGs to identify those who were active, and to verify if members were still living in the same geographical area, or if some had moved: “*Now, what we should do ( …) is to revitalize the CAGs, truly check who is inside [the CAG]. They have to be from the same neighbourhood ( …) because the CAG is very important.”* (FGD 1, health care providers, nurses).

To properly assess the CAG model of care, providers proposed various strategies, including: *“taking turns to visit a different neighbourhood every month, to talk with the group and find out how things are going, and if people are adhering or not. Because even though some had no contact [with the clinic], there are always a few who know about the others.”* (FGD 1, clinical officer).

Providers believed that relevant information about the whereabouts of patients can be adequately assessed through the social network of CAG members, even if some had lost contact with the clinic.

The role of the focal point was also discussed as something which may need to change: *“Some focal points of some groups will need to be replaced ( …). We may need to meet with the group to identify another focal point, who is a bit more dynamic, and who really supports the adherence of the group.”* (FGD 2, health care providers, nurse). They agreed that a focal point had to be *“a person who is the pillar of that group with regards to treatment”* (FGD 5, health care providers, counsellors).

## Discussion

This is the first study that explored how changing life circumstances can affect peer-led ART delivery. Participants reported different experiences that can interrupt or challenge the successful functioning of the CAGs. The main themes were mobility of CAG members due to employment, conflict and changes in relationships between group members and alcohol use. Moreover, treatment adherence and treatment response, for instance in terms of virological suppression, may change over time. The CAG dynamic differed between individual groups. Some CAGs were unsuccessful in offering support to other members when non-adherence was identified but not reported, yet in other cases CAG members were reported to intensify and find creative ways to support a fellow member. Providers agreed that more emphasis should be placed on the support of the processes required to ensure a successful running of the CAG model.

When the CAG model was introduced in Tete, it was presented as an approach responding to a concrete patient need [5]. Some activities, like monthly ART collection from health facilities, were constant across CAGs. Other activities, like monthly meetings and distribution of ART within the community after collecting a refill at the health facility, were adapted to the daily realities and preferences of the group members, explaining why the dynamic differed between groups. Moreover, our findings show that people’s lives are not infinitively stable, especially in a rural context with high rates of migration. Hence, ART delivery that relies on a social network of PLWH for its daily functioning should be flexible and adaptive, not only when CAGs are formed, but thereafter.

However, installing flexible and adaptive care delivery systems for lifelong treatment can be challenging [17]. Health-care providers are mostly used to implementing standardized guidelines, especially in primary health care settings where task shifting allowed engaging a lower cadre in clinical HIV care [18]. When CAGs were rolled out nationally in Mozambique in 2011, [19] guidelines and standard operating procedures were needed to ensure that health-care providers all understood the model and how it should be implemented [19]. The inherent risk was the instalment of a “one size fits all” approach to CAG roll out and monitoring which was not adapted to different populations. However, guidance on community participation for such a differentiated model of care delivery should ensure patient- and community-catered approaches to identify needs, design the strategy and agree on roles for the different stakeholders [20].

To ensure adaptive implementation and modifications of the CAG dynamic to the changes in life circumstances of its members, feedback loops should be installed between the CAG and the health facility care providers [17]. During meetings these stakeholders could discuss identified challenges and adjustments to the CAG dynamic, and also disseminate lessons learnt from innovative approaches in other communities. During the early years of CAG implementation in Tete this iterative process engaged patients in the unprecedented role of co-designer of ART delivery strategies [7].

This study has some important limitations. Firstly, only patients retained in care were interviewed, thus the views of those lost to follow-up are not included in our research when they are, arguably, also an extremely vulnerable group. Moreover, interviewees may have associated the researchers with health-care providers, which may have introduced a degree of social desirability bias. To mitigate this type of bias, interviews and focus group discussions with CAG members were organized outside health facilities, at venues identified by patients. Secondly, most interviews were conducted in the presence of a non-Mozambican researcher, which to some extent may have introduced further bias. We did not explore all identified codes separately for “classic” and “family” CAGs. Finally, even though findings from this research may not be directly transferable to other settings, they are important as they may inform policy makers and researchers in various contexts who work with long-lasting peer groups. Our research also had important strengths. Interim analysis was continuous, to agree on when saturation for themes explored in previous IDIs and/or FGDs was reached, to identify emerging themes and to inform the topic guides with open-ended questions. Finally, study participants included CAG members and providers, which allowed for the triangulation of data collected from both groups.

## Conclusions

We described how changing life circumstances, such as travel and employment, changes in relationships, as well as a lack of participation by CAG members modified the CAG dynamic in Tete, Mozambique. In some instances, this led to non-adherence and loss to follow-up. However, some CAGs responded to these challenges by intensifying peer support, including to those diagnosed with virological failure. It remains unclear, however, how flexible community participation for an essential medical task such as ART delivery can be organized and sustained, and how much responsibility and decision-making with regards to the distribution of antiretrovirals and the strengthening of the peer dynamic can be delegated to patient-led peer groups. A “one size fit all” approach to CAG implementation is neither possible nor desirable, as people and groups are unique, and have changing circumstances which need to be recognised and appreciated within differentiated models of care such as CAGs.

## Supplementary Information

**Additional file 1.** IDI and FGD Interview Guide

## Data Availability

The transcripts generated during and analysed during the current study are not publicly available to protect the confidentiality of study participants, but are available from the corresponding author on reasonable request, and after seeking permission of relevant Ethics Review Boards.

## Abbreviations

ART: antiretroviral therapy
CAG: Community ART Groups
IDI: in-depth interviews
FGD: focus group discussion
IQR: interquartile range
PLWH: People Living With HIV
WHO: World Health Organization
