# Supplementary material for: “I don’t know when he will be back”: life-changing events challenge the community ART Group model– a qualitative research study, Tete, Mozambique
Source: BMC Public Health. 2021 Nov 4;21:2004. doi: 10.1186/s12889-021-12087-8 (PMC8567643; doi:10.1186/s12889-021-12087-8)
Supplement: Supplementary file 1 — Additional file 1. IDI and FGD Interview Guide [file 12889_2021_12087_MOESM1_ESM.docx]

**Supplementary file: IDI and FGD Interview Guide**

**IDI Interview Guide**

| IDI Information Guide – CAG/IC**^‡^** patient | |
| --- | --- |
| Participant IDNO \|__\|__\|__\|__\| Gender Male / Female Researcher Initials \|__\|__\|__\|  Health facility number \|__\|__\| Date \|__\|__/__\|__/__\|__\|  **Introduction**  I am ______________________________ from ______________________   - General purpose of the study - Aims of the interview and expected duration - Who is involved in the process (other participants) - Why the participant’s cooperation is important - What will happen with the collected information and how the participant/target group will benefit - Any questions? - Consent   **Warm up [demographic & work history]**  Can I ask some details about you and your job?  Job Title ____________________________  Highest Educational Grade attained ___ __ Year of graduation____________  Years worked at this facility \|__\|__\|yrs\|__\|__\|mths  Are you originally from this area/district? □ Yes □ No  How old are you? □ Under 30yrs □ 30-40yrs □ Over 40yrs  Do you have any children over 5yrs old? □ Yes □ No  Do you have any children under 5yrs old living with you now? □ Yes □ No  **Now I am going to ask you some questions about your experiences with the ARV therapy.** | |
|  | **Topics and Prompts** |
| **1** | *How important is for you to have a regular VL^1^? Why?*  **Prompts**: good health, virological suppression, long life with HIV, monitor |
| **2** | *What do you know about virological failure (VF)?*  **Prompts**: explained by HP^2^, previous viral load, previous results, what it happens if VL is high, becoming sick, unable to work, I want to learn more. |
| **3** | *What does VF^3^ mean to you?*  **Prompts**: relationship w/family, friends, HPs, peers; morale: guilt, angry, pressure from familiars, frustrated, emotions, changing adherence strategy. |
| **4** | *How do you feel in taking treatment every day for life?*  **Prompts**: self-awareness on HIV+ status; changes over time, relationship, impossible, no problem, being pregnant, co-infection with TB |
| **5** | *What means adherence to you?*  **Prompts**: explained by HP, daily pill intake punctuality; difficult in taking daily pills; difficulties: traditional treatment, nutrition; hide pills intake, secrecy of HIV status, , recovered health status; side effects; pregnancy; alcohol intake; confusion between treatments; pill burden w/different treatments (TB), resistance, drugs supply. |
| **6** | *Do you know about EAC^4^? If YES what do you think about it?*  **Prompts:** compliances appointments, changes in adherence. |
| **7** | *How do you organize your daily pill intake?*  **Prompts**: personal strategies; out of routine situations: funeral, travel, hospital children intake, work; support family, cell phone; community leader, CAG^5^; not taking them. |
| **8** | *What do you think about treatment interruptions, please explain?*  **Prompts**: feeling well, good, saving money, make money (selling); worried about it, effect on health, health provider’s attitude, family/CAG pressure. |
| **9** | *Do you know about CAG? If YES what do you think about it? (for non CAG members)*  **Prompts:** information sharing in the community; information by counsellor, support; confidence in your community, risks for stigma, convenient. |
| **10** | *Are you participating in CAG? How do you experience that? (For CAG members)?*  **Prompts**: usefulness for daily intake; help, support, peer pressure, conflict, changes in community meetings overtime. |
| **11** | *Is there anything related to the subject we have not discuss yet?* |
| **Closing**  Is there anything else you think is important about Viral Load and/or Virological Failure that we have not talked about?   - Summarize - Thank participant   Provide extra information and contacts to participants | |

*^‡^IC: Individual Care; ^1^VL: Viral Load; ^2^HP: Health Provider, ^3^VF: Virological Failure; ^4^EAC: Enhanced Adherence Counselling; ^5^CAG: Community Adherence Group*

**FGD Interview Guide**

| FGD Information Guide - HP/CAGFC^‡^  Participant IDNO \|__\|__\|__\|__\| Researcher Initials \|__\|__\|__\|  Health facility/Meeting local name \|__\|__\|__\|__\|__\|__\|__\|__\| Date \|__\|__/__\|__/__\|__\|  **Introduction**  I am ______________________________ from ______________________   - General purpose of the study - Aims of the interview and expected duration - Who is involved in the process (other participants) - Why the participant’s cooperation is important - What will happen with the collected information and how the participant/target group will benefit - Any questions? - Consent   **Now I am going to ask you some questions about your experiences with the ARV therapy.** | |
| --- | --- |
|  | **Topics and Prompts** |
| **1** | How important is to have a regular VL^1^ for patients on ART**^†^**? Why?  **Prompts**: good health, virological suppression, monitor, importance perceived by patients |
| **2** | How do patients with high VL understand virological failure?  **Prompts**: role HP,^2^ explanation HP, what do patients say, do patients ask questions |
| **3** | How do patients react when they have a high VL?  **Prompts**: don’t care, guilt, angry, frustrated, pressure from family, change adherence strategy, relationship HP – patient |
| **4** | What may affect taking the patient’s attitude to a lifelong daily treatment, like ART?  **Prompts**: self-awareness on HIV+ status; secrecy of HIV status; hide pills intake; explanation by HP; confusion between treatments; TB; traditional treatment, nutrition; side effects; alcohol intake; changes over time: feeling better, divorce, children, work; out of routine situations: funeral, travel; pressure of family/ CAG;^3^ stigma; drugs supply; relation HP-patients; impossible; what do patients say |
| **5** | Which practical adherence strategies for daily pill intake do patients with a high VL use?  **Prompts**: personal strategies; support family, cell phone; community leader, CAG; not taking them. |
| **6** | Why there are some many patients with a high VL?  **Prompts:** support pill intake, information sharing in the community; stigma, supply drug, CAG |
| **7** | How affects EAC daily pill intake in patients with a high VL?  **Prompts:** compliance to appointments, changes in adherence, does it work - why, re-suppression |
| **8** | How did support for pill intake in the CAG evolve over time?  **Prompts:** community meetings, delivery, support pill intake, information sharing in the community; stigma, |
| **9** | Is there anything related to the subject we have not discuss yet? |
| **Closing**  Is there anything else you think is important about Viral Load and/or Virological Failure that we have not talked about?   - Summarize - Thank participant   Provide extra information and contacts to participants | |

*^‡^CAGFC: Community Adherence Group Focal Point; ^†^ART: Antiretroviral Therapy*

*^1^VL: Viral Load; ^2^HP: Health Provider, ^3^CAG: Community Adherence Group*
